# Resonance and beat perception of ballroom dancers: An EEG study

**DOI:** 10.1371/journal.pone.0312302

**Published:** 2024-10-21

**Authors:** Xuru Wang, Chenglin Zhou, Xinhong Jin

**Affiliations:** 1 Shanghai Institute of Early Childhood Education, Shanghai Normal University, Shanghai, China; 2 School of Psychology, Shanghai University of Sport, Shanghai, China; 3 Key Laboratory of Motor Cognitive Assessment and Regulation, Shanghai, China; 4 Key Laboratory of Exercise and Health Sciences (Shanghai University of Sport), Ministry of Education, Shanghai, China; University of Turin, ITALY

## Abstract

**Purpose:**

The ability to synchronize the perceptual and motor systems is important for full motor coordination and the core determinant of motor skill performance. Dance-related training has been found to effectively improve sensorimotor synchronization, however, the underlying characteristics behind these improvements still warrant further exploration. This study was conducted to investigate the behavioral and neuroactivity characteristics of ballroom dancers relative to those of non-dancers.

**Participants and methods:**

Thirty-two dancers (19.8 ± 1.8 years old) and 31 non-dancers (22.6 ± 3.1 years old) were recruited to perform a finger-tapping task in synchrony with audiovisual beat stimuli at two intervals: 400 and 800 ms, while simultaneously recording EEG data. Behavioral and neural activity data were recorded during the task.

**Results:**

The dancers employed a predictive strategy when synchronizing with the beat. EEG recordings revealed stronger brain resonance with external rhythmic stimuli, indicating heightened neural resonance compared to non-dancers (*p* < 0.05). The task was more challenging with an 800-ms beat interval, as observed through both behavioral metrics and corresponding neural signatures in the EEG data, leading to poorer synchronization performance and necessitating a greater allocation of attentional resources (*ps* < 0.05).

**Conclusion:**

When performing the finger-tapping task involving audiovisual beats, the beat interval was the primary factor influencing movement synchronization, neural activity and attentional resource allocation. Although no significant behavioral differences were observed between dancers and non-dancers, dancers have enhanced neural resonance in response to rhythmic stimuli. Further research using more ecologically valid tasks and stimuli may better capture the full extent of dancers’ synchronization abilities.

## Introduction

Perceptual–motor interaction plays a crucial role in the functioning of the human brain, with multiple cortical areas involved in perceptual recognition and motor execution [1]. When music abruptly begins, individuals naturally respond by spontaneously syncing with the external rhythm, engaging in activities such as rhythmic head movements, arm waving and overall body motion [2, 3]. This represents a fundamental aspect of human behavior when responding to rhythmic stimuli, showcasing an essential aspect of our interaction with external beat. In such contexts, success or failure can hinge on mere fractions of a second. An essential aspect of motor skill development is the effective integration of synchronized signals from the external world. Among various activities, ballroom dance stands as a form of competitive sports that merges athleticism with the art of dance. It is among the activities where the coordination between external stimuli and bodily movements is most pronounced, playing a significant role in enhancing an individual’s motor coordination skills. Ballroom dancers exemplify the need for coordination between body movements and musical rhythmic stimuli, which entails a series of processes related to beat perception and movement execution. The representation of movement intertwines the perceptual and motor systems, allowing for the coordination and adjustment of external stimulus perception and movement processes [4]. An understanding of the interplay between perceptual and motor processes in the dancers’ brain has significant implications for motor coordination, motor training and rehabilitation.

Sensorimotor synchronization (SMS) is a critical element in the framework of perceptual–motor interaction and dancers’ coordination with external musical rhythm and movement [5, 6]. It facilitates the harmonization of individual actions with external stimuli, reflecting the intricate interplay between individuals and their surrounding environments. During SMS, individuals’ action responses and the sensory stimuli they encounter typically exhibit periodic variations [5, 7]. Individuals often derive predictive cues from such rhythmic cycles. The synchronization of temporal rhythms significantly influences perceptual–motor coordination and serves as a fundamental basis for various everyday movements and activities associated with music [8–10]. The finger-tapping task has been the paradigm used most commonly to investigate SMS [5, 7], but the advanced synchronization capabilities of dancers offer a rich area for further exploration, which is both a current and pressing research avenue.

Researchers have dedicated significant efforts to the unravelling of the intricate processes through which the brain receives and processes sensory input, generates motor output, and integrates input and output information to achieve efficient SMS [11–14]. Moreover, SMS underscores the influence of endogenous ‘top-down’ predictive and adaptive mechanisms on beat perception and the selection of appropriate responses. These processes entail the adjustment of the phase and period of internal oscillations in response to external stimuli [15]. In dance movement, the motor and sensory systems intricately intertwine within the representation of actions to coordinate the process of perceiving and tracking external rhythms. Dance training can enhance neuroplasticity by integrating activity across multiple brain regions [16], improving perceptual-motor coordination performance, and exhibiting domain-specific characteristics [17–19].

Ballroom dance involves not only the fusion with musical rhythms but also a series of coordinated movements that engage a multitude of perceptual motor processes associated with beat perception and motor execution. From an individual experiential perspective, dance movement is inherently linked to the processing of musical rhythms. However, current research indicates differences in rhythm information processing between music and dance training. Musicians demonstrate advantages in auditory rhythm discrimination and tapping synchronization, while dancers exhibit strengths in visual-auditory integration and physical imitation [17–19]. Other studies suggest that, compared to visual rhythms, dance experience can further enhance temporal processing of auditory rhythms [20] and SMS [21]. Hence, while dance movement is closely related to musical rhythms, dance training may present specialized advantages in beat perception and action synchronization compared to music training. Previous research has predominantly focused on the impact of musical experience on perceptual-motor coordination, leaving a relative dearth of studies on the coupling process of perceptual-motor coordination and the specialized advantages within dance-specific training, especially in competitive sports dance movement. This unique characteristic plays a significant role in the enhancement of individuals’ coordination abilities during movement [20, 22]. A recent study showed that college-level expert ballroom dancers exhibited heightened sensitivity to sensory stimuli and performed exceptionally well in SMS tasks relative to general university students, enabling synchronization across a broader range of rhythmic frequencies [21]. However, how ballroom dance experience facilitates the perceptual-motor coupling process in SMS remains unknown. Previous studies have demonstrated that audiovisual stimuli result in more accurate and stable SMS compared to auditory or visual stimuli alone [5, 21], and we found individuals with dance experience exhibit distinct behavioral characteristics when responding to auditory and visual stimuli compared to control groups [21]. To investigate the effects of different rhythm frequencies on SMS and the associated neural activity in individuals with dance experience, we used audiovisual stimuli to avoid the potential confounding effects of unimodal advantages. Moreover, audiovisual stimuli enhance the ecological validity of our study, as real-world synchronization tasks often involve multiple sensory modalities.

The integration of input from the perceptual system and the output of the motor system can be elucidated through the examination of neural oscillatory activity [15]. Neural resonance theory [23, 24] posits that neural oscillations synchronize with external rhythmic stimuli, facilitating perceptual and motor processes. For instance, when listeners are exposed to a sequence of tones at 2.4 Hz, the electroencephalographic (EEG) response reveals a component at the same frequency [25]. This synchronization is thought to enhance the brain’s ability to process and anticipate rhythmic patterns, which is crucial for activities such as music perception and sensorimotor synchronization. When the oscillation frequency falls within the synchronization boundary and the coupling strength is sufficient, neural oscillations align with the frequency of external stimuli, resulting in synchronization effects. According to neural resonance theory [23, 24], rhythmic frequencies and beats correspond to synchronized neural rhythms that mirror acoustic rhythms. This mechanism influences various aspects of cognition, including temporal anticipation, attention and motor coordination [26]. During beat perception, neural oscillations in the brain dynamically adapt to changes in external rhythmic patterns, indicating that resonance between the two is the foundation for motor coordination. Specifically, neural responses elicited by stimuli are associated with delta band oscillations, and the phase alignment of these oscillations serves as the foundation for temporal prediction, even in the absence of rhythmic stimuli. On the other hand, high-frequency beta oscillations are associated with motor functions, establishing connections with rhythmic stimulus sequences, participating in the encoding of rhythmic beats, while also predicting the temporal characteristics of sound rhythms [27, 28]. The Action Simulation for Auditory Prediction [29] hypothesis proposes that auditory-motor facilitation is achieved through bidirectional signal transmission between the dorsal auditory stream and the motor planning system, emphasizing the role of the motor system in forming an internal predictive model in beat perception. Although the pivotal role of neural oscillations in perceptual–motor coupling has been confirmed, further exploration is warranted to understand how neural oscillatory activity regulates bottom-up (e.g., responses to external stimuli) and top-down (e.g., prediction and adaptation) processes [30, 31].

Event-related potentials (ERPs) serve as a valuable tool for the investigation of the brain’s predictive processes, as they reflect specific responses within narrow temporal windows. They contribute to the understanding of predictive mechanisms, including the enhancement of predictability through increased repeatability and prediction [32]. The N1 component, a negative deflection in the ERP waveform, plays a crucial role in prediction and attention. Its peak typically occurs 100–200 ms after stimulus onset [27, 33, 34]. The P2 component is sensitive to recognition and can be influenced by the cognitive load [35]. The neural representation of oscillations in SMS processes, particularly in the context of sports experience, requires further investigation.

The primary aim of this study was to understand how different rhythmic contexts influence SMS performance and neural responses in individuals with varying levels of dance training. We further explored the neural activity characteristics underlying the SMS process of dancers (relative to non-dancers) using audiovisual rhythmic stimulation with different beat intervals, which is usual in dance performance and daily contexts. This includes a deeper exploration of how dance training, particularly in comparison to other activities involving movement synchronization with music, contributes to neural elements of beat perception. We hypothesized that dancers’ SMS performance would be better than nondancers, meanwhile, dancers would exhibit distinct neurophysiological responses. Specifically, we expected that dancers would demonstrate greater resonance power in delta band frequencies and more pronounced top-down prediction in response to external rhythmic stimuli.

## Material and methods

### Participants

The sample size was computed by an a priori power analysis using G*Power 3.1 [36] by selecting ‘F tests-ANOVA: Repeated measures, within-between interaction’ as statistical test, with effect size partial eta-squared = 0.25, α err prob = 0.05, power (1 - β err prob) = 0.90. Specifically, we derived the effect size from our previous findings [21], which reported average effect size of 0.25 in a similar behavioral paradigm [37]. The calculation results indicated that a sample size of 46 participants was required for this study. To enable statistical assessment with a desired effect size, EEG recordings and finger-tapping data were collected from a total of 63 participants [32 ballroom specialists (10 males, mean age 19.8 ± 1.8 years) and 31 non-dancers (10 males, mean age 22.6 ± 3.1 years)]. All participants were of Chinese nationality. The dancers had an average dance training period of 8.5 ± 3.7 years and average weekly dance training time of 10.78 ± 1.96 h. Prior to participation, all individuals confirmed that they had normal hearing and normal or corrected-to-normal visual acuity and that they were right-handed. No participant reported any history of a disorder that could potentially impact task performance. The ethics committee of Shanghai University of Sport approved the experimental protocol with the Declaration of Helsinki (102772022RT080) and all participants have written the informed consent. For the present study, participant recruitment commenced on October 1, 2022 and concluded on December 31, 2022. The duration of the recruitment period was three months to ensure a sufficient number of participants for the analysis.

### Stimuli

The study was conducted in a dimly lit and acoustically isolated chamber in a psychology laboratory. The experimental protocol was developed and implemented using Psychotoolbox 3.0 in Matlab (2013a), with visual and auditory stimuli provided via a desktop computer (X64, Windows 7). Synchronous audiovisual stimulation refers to the simultaneous presentation of auditory and visual stimuli. The visual stimulus consisted of a blue circle with a diameter of 2.5 cm that flashed evenly in the centre of a grey background. The auditory stimulus was a low-pitched (500-Hz) pure-tone ‘beep’ with a duration of 40 ms, and were presented with in-ear headphones (Sennheiser, CX 3.00) at a comfortable hearing level. The audiovisual stimulation presented synchronously and beat interval encompassed frequency of 400 and 800 ms. The display screen was a 19-inch monitor with a resolution of 1440 × 900 and refresh rate of 60 Hz. The auditory stimulation was delivered through in-ear headphones, with the volume adjusted to suit individual subjects’ loudness perception.

### Procedures

**Fig 1:**
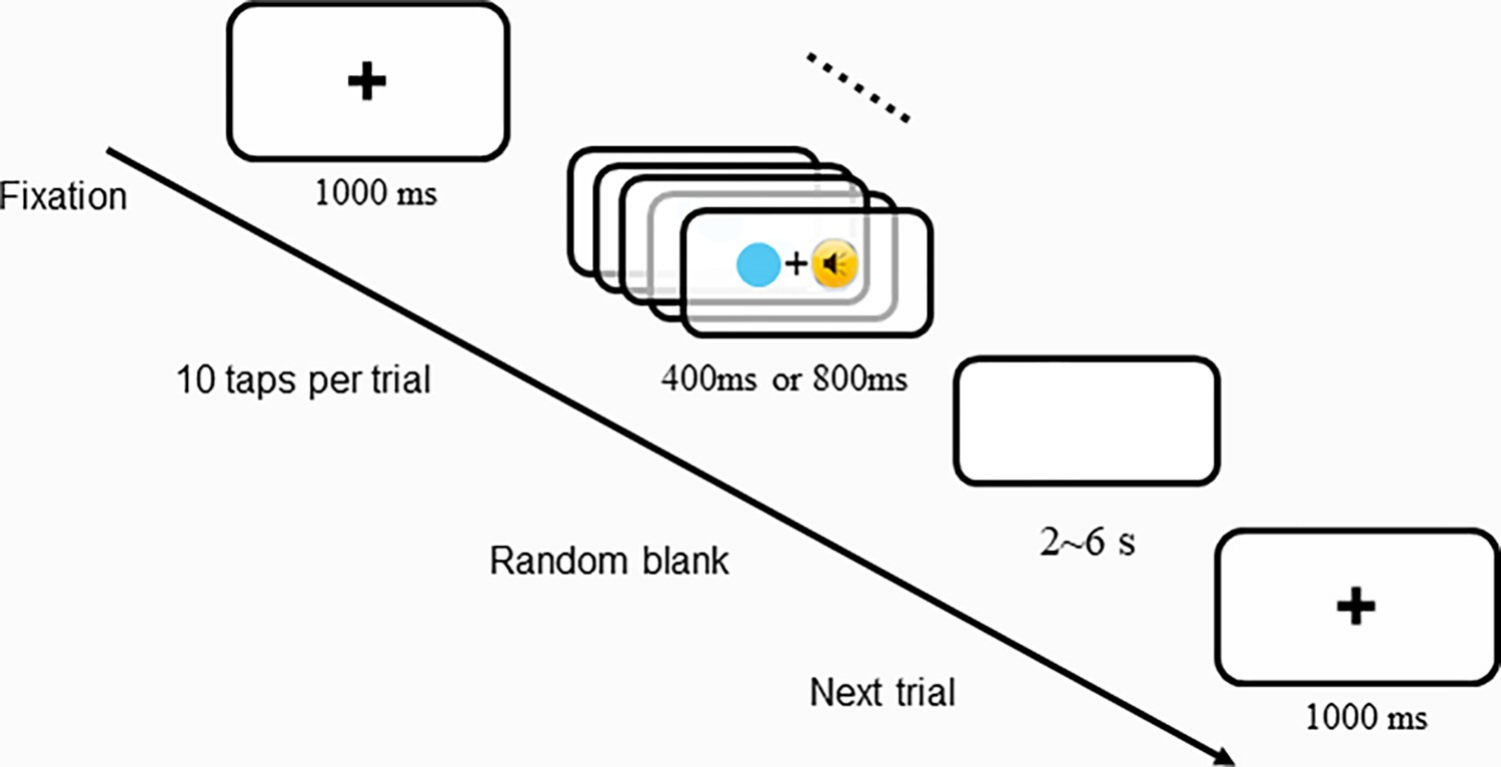
Schematic of the experimental procedure. The experimental sequence commences with the presentation of a fixation point (’+’) on the screen for 1000ms. Subsequently, a series of audiovisual beat stimuli (consisting of a flashing dot and pure auditory tones) are continuously displayed. Participants are instructed to perform synchronous tapping by pressing the ’M’ key on the keyboard in response to the presented beat for 10 taps. The task requires participants to synchronize their tapping as closely as possible with the appearance of the rhythmic stimuli for optimal performance. The audiovisual beat intervals (400ms and 800ms) are presented randomly throughout the task.

Each participant filled out a preliminary information form and was then instructed to assume a comfortable seated position at a distance of 1 m from the computer screen. Following a thorough explanation of the experimental procedure and task instructions, the participant donned a brain cap and in-ear headphones for the SMS tasks. Prior to the commencement of each trial, a visual fixation point (‘+’) was presented at the centre of the display screen for 1000 ms. The participant was instructed to use their index finger to press the ‘M’ key on the keyboard to tap in sync with the audiovisual beats. The stimuli were presented a total of 10 times per trial with fast (400-ms) and slow (800-ms) beat intervals randomly, resulting in a total tapping duration of 4 seconds for the 400ms interval and 8 seconds for the 800ms interval. Participants were instructed to tap in synchrony with the stimuli, aiming for 10 taps per trial. The interval between trials ranged randomly from 2 to 6 seconds (Fig 1). The experiment consisted of 3 blocks of 20 trials each, with total 30 trials for each beat intervals.

### EEG data recording

EEG signals were captured using a 64-channel electrode cap configured according to the international 10–20 system with Ag/AgCl electrodes and an EEG recording and analysis system (Brain Products, brainAMP system, Germany) with a bandwidth ranging from 0.01 to 100 Hz and a sampling rate of 1000 Hz. To monitor horizontal and vertical eye movements, electrodes were placed 1 cm from the outer orbital surface of the right eye and inferior orbital surface of the left eye to record the horizontal and vertical electrooculograms, respectively. The impedance of the electrodes was maintained consistently below 5 kΩ to ensure optimal signal quality.

### Data processing

Following previous research [13, 21], synchrony and asynchrony were assessed by subtracting inter-stimulus intervals from inter-tap intervals. The standard deviation of tapping asynchrony was used to estimate the variability in SMS over time [13, 38]. To compute the absolute mean asynchrony, we first calculated the asynchrony for each trial, then converted the asynchrony values of each trial into absolute values, and at last, computed the mean of these absolute values for each participant. This measure indicated the accuracy of synchronization with mitigation of the influence of predictive (negative values) and tracking (positive values) strategies on SMS tasks [39, 40].

The pre-processing of the EEG data was performed using frequency domain and ERP analyses. The frequency domain analysis was conducted using the Brain Vision Analyzer 2 (version 2.1, Brain Products GmbH). The average ear mastoid (TP9 and TP10) potential was used as a reference, and the frontal-central (FCz) electrode was restored. A series of filtering procedures (main power removal at 50 Hz, high-pass filtering at 0.1 Hz and low-pass filtering at 30 Hz with a slope of 24 dB/octave) was applied. Ocular correction was conducted on the filtered EEG data by referring to the two electrooculogram channels. Independent component analysis was performed to eliminate ocular electrical artifacts. Artifacts exceeding the amplitude threshold of ±100 μV were automatically rejected.

In order to reveal the time and frequency changes of non-stationary EEG signals, time-frequency analysis techniques were used. MATLAB software and EEGLAB toolbox were used for EEG time-frequency processing. We segmented based on trial signals, with −200ms to 0 before the first stimulus presentation as the baseline-correction period. According to the topographical maps for each condition in two groups and previous rhythm perception studies [27, 41], the analysis was conducted in frontocentral electrode cluster (Fz, FC1, FCz, FC2, Cz). The time-domain waveforms of all single trials under a certain electrode were first subjected to continuous wavelet transform on each EEG channel using the ‘*cmorl-1*.*5*’ complex wavelet function in MATLAB [42], which with a centre frequency of 1 Hz, spectral bandwidth of 1.5 Hz, linear frequency ranging from 0.5 to 30 Hz and frequency resolution of 0.05 Hz. And then the spectral power was obtained using the periodogram method. Then, corresponding power can be computed as:

$$Power(A_{c,:})=log10[CWT{\left(A_{c,:}\right)}^{2}/F]$$ (1)

Where CWT(A_c,:_) represent the wavelet coefficients at given discrete time series on the cth channel. Finally, the time-frequency distribution of the power under a certain electrode was obtained by superposition averaging and normalization. Power calculations were performed for the 1.25-Hz, 2.5-Hz peak power and delta (1–4 Hz) frequency bands, which represent characteristic neural oscillatory activity in response to rhythmic stimulation in the brain [15, 27].

The frontocentral electrode cluster was chosen specifically for the analysis of ERP components elicited by the audiovisual beat stimulation. ERP data elicited by the stimuli were segmented from 100 ms before to 400 ms after the onset of each stimulus, with the onset serving as the reference point (0 ms), under the 400-ms and 800-ms interval conditions. Baseline correction was performed by referencing the 100 ms preceding the reference point. The segmented data were then processed using the MATLAB software (version 2012b; The Mathworks, Inc., Natick, MA, USA) and EEGLab toolbox. These tools were employed to superimpose and analyse the exported data for the extraction of the peaks of N1 and P2 components. The time windows for the N1 and P2 components’ peak evoked by audiovisual rhythmic stimulation were defined as 100–170 and 170–250 ms, respectively.

To assess the effect of the beat interval on SMS, repeated-measures analysis of variance [ANOVA; 2 (group) × 2 (beat interval)] was conducted using the SPSS 17.0 software. Data from the dancer and non-dancer groups were analyzed. The assumption of sphericity (homogeneity of variance) was evaluated using Mauchly’s test. When this assumption was violated, the Greenhouse-Geisser correction method was applied. The threshold for statistical significance was set at *p* < 0.05.

To further examine the relationship between behavioral performance and neurophysiological responses, we conducted a correlation analysis between tapping asynchrony and neural oscillations. Pearson correlation coefficients were computed to evaluate the relationship between these behavioral and neural measures. Then, separate analyses were performed for each group (dancers and non-dancers).

## Results

### Behavioral results

#### Mean asynchrony

**Fig 2:**
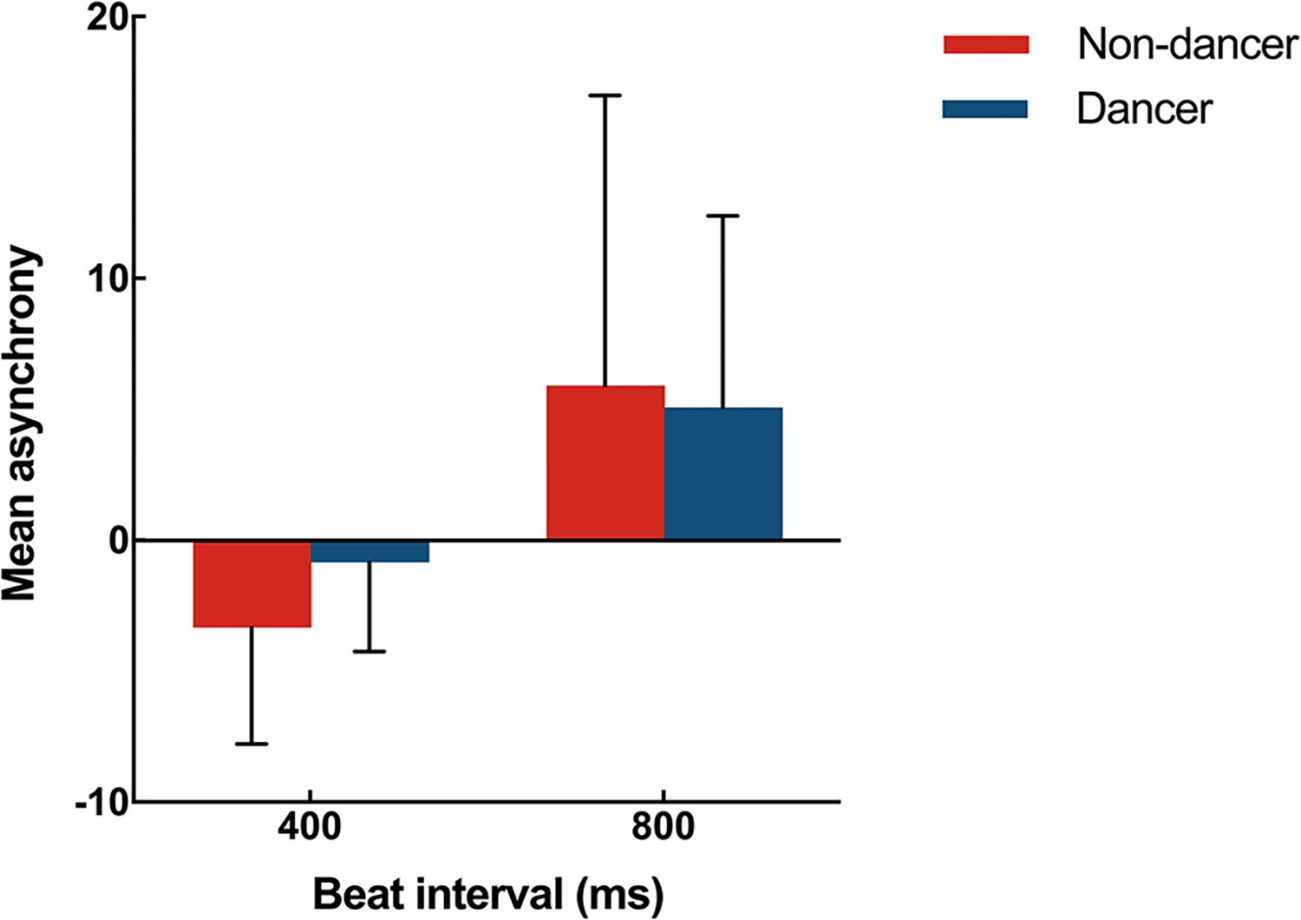
Mean asynchrony of dancers (solid lineblue) and non-dancers (dashed linered) with 400- and 800-ms audiovisual beat stimuli. Error bars indicate standard errors.

At the 400ms beat interval, there was no significant difference in mean asynchrony values between dancers (*Mean±SD*, -0.83±19.35 ms) and non-dancers (-3.34±24.72 ms), indicating that both groups demonstrated negative synchronization, with key presses occurring before rhythmic stimulus onset. The mean asynchrony values (± SD) at the 800ms beat interval for dancers and non-dancers were 5.07 ms (± 41.47 ms) and 5.93 ms (± 61.66 ms) respectively, which also showed no significant differences (*p*>0.05). At the 800-ms condition, average response times lagged behind rhythmic stimulus onset, indicating a delay in synchronization, in both groups (Fig 2).

#### Absolute asynchrony

The repeated-measures ANOVA revealed a significant main effect of the beat interval [*F* (1,61) = 45.51, *p* < 0.0001, *η*_p_^2^ = 0.427]. Specifically, the absolute difference in asynchrony was significantly smaller for the 400-ms interval than for the 800-ms interval, reflecting greater synchronization accuracy (15.90 ± 15.24 vs. 41.30 ± 31.58 ms, *p* < 0.0001). The main effect of group was not significant [*F* (1,61) = 3.51, *p* = 0.07], although the absolute asynchrony value was lower for dancers than for non-dancers (24.15 ± 18.23 and 33.19 ± 27.17 ms, respectively; *p* = 0.06), suggesting a trend toward greater accuracy in tapping to the beat in the dance group. No significant interaction between the group and beat interval was detected [*F* (1,61) = 3.27, *p* = 0.08].

#### Standard deviation

The ANOVA revealed a significant main effect of the beat interval on the standard deviation of asynchrony [*F* (1,61) = 24.58, *p* < 0.0001, *η*_p_^2^ = 0.287], which was significantly smaller, reflecting more synchronization stability, with the 400-ms beat interval than with the 800-ms interval (*p* < 0.0001). The main effect of the group was not significant [*F* (1,61) = 0.14, *p* = 0.71] and no significant interaction between the group and beat interval was detected [*F* (1,61) = 0.06, *p* = 0.81], suggesting that the synchronization stability did not differ between groups across the beat intervals.

### EEG results

#### Resonance power

Based on the resonance theory [23, 24], the beat frequency is associated with neural oscillatory synchrony, which has the potential to enhance temporal expectation, attention and coordination. We analysed the resonance power at different beat frequencies.

*1*.*25 Hz*. We examined the frequency point power at the frontocentral clustering sites across various beat intervals to investigate the presence of resonance activity elicited by different stimuli. The ANOVA revealed a significant main effect of the beat interval on resonance activity at 1.25 Hz [*F* (1,61) = 160.098, *p* < 0.0001, *η*_*p*_^2^ = 0.724], which was significantly greater with the 800-ms interval than with the 400-ms interval (*p* < 0.0001). The group also had a significant effect [*F* (1,61) = 12.749, *p* = 0.001, *η*_p_^2^ = 0.173], with dancers exhibiting significantly more power at 1.25 Hz than did non-dancers (*p* = 0.001). No significant interaction between the group and beat interval was detected [*F* (1,61) = 0.391, *p* = 0.534].

*2*.*5 Hz*. The ANOVA revealed a significant main effect of the beat interval on the power at the frontocentral sites at 2.5 Hz [*F* (1,61) = 93.055, *p* < 0.0001, *η*_p_^2^ = 0.604], with significantly more activity observed with the 800-ms beat interval than with the 400-ms interval. No significant main effect of the group effect [*F* (1,61) = 2.197, *p* = 0.143] or interaction [*F* (1,61) = 0.0002, *p* = 0.990] was detected.

*Delta band*. The ANOVA revealed a significant main effect of the beat interval on the average evoked delta power [*F* (1,61) = 328.393, *p* < 0.0001, *η*_p_^2^ = 0.843], which was significantly greater, reflecting more activation, with the 800-ms interval than with the 400-ms interval (*p* < 0.0001). The group also had a significant main effect on the delta power [*F* (1,61) = 5.759, *p* = 0.019, *η*_p_^2^ = 0.086], with dancers exhibiting significantly greater delta energy than non-dancers during rhythm synchronization. No significant interaction between the group and beat interval was observed [*F* (1,61) = 0.017, *p* = 0.897].

### ERP results

#### N1 component

**Fig 3:**
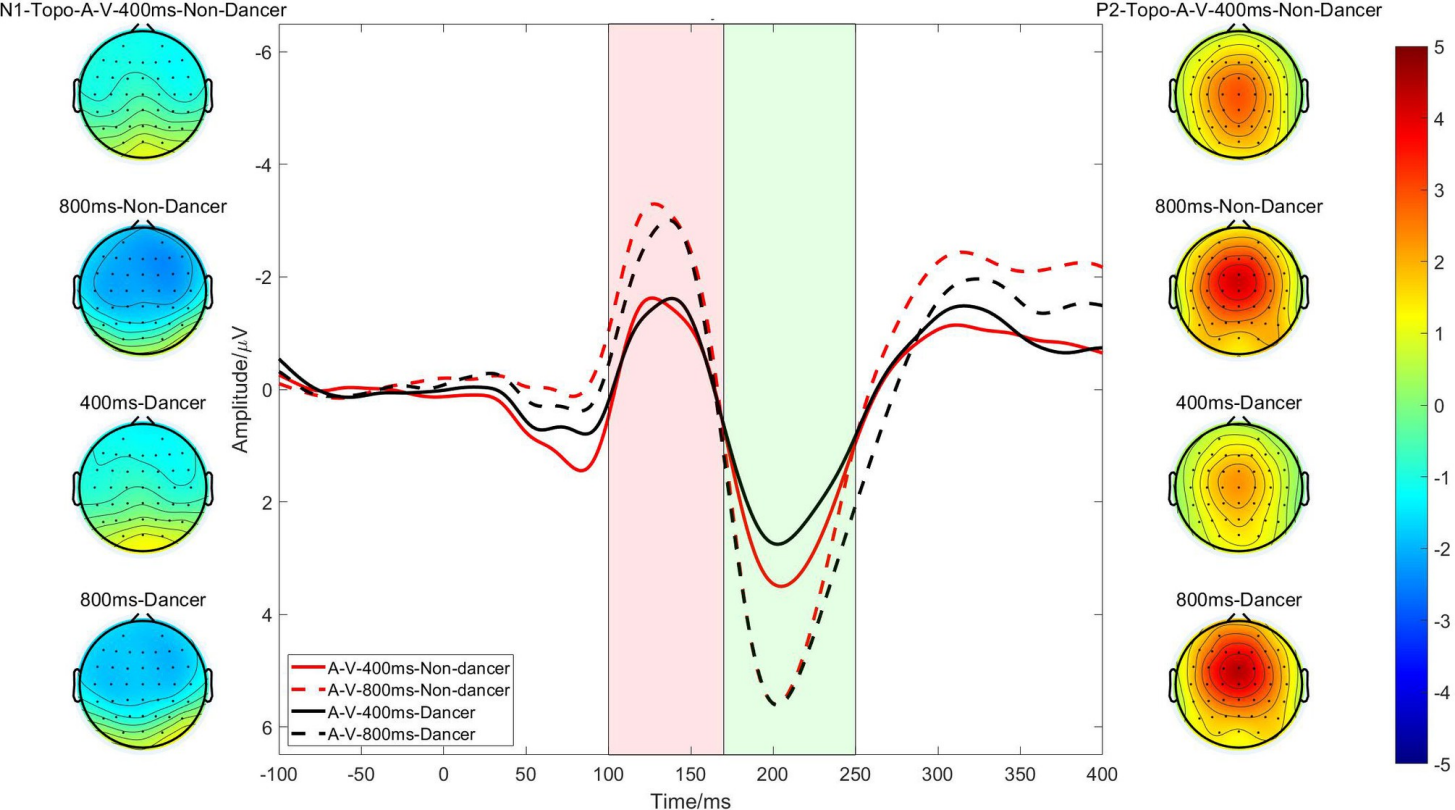
Event-related potentials at the frontocentral electrodes at the onset of 400-ms (solid line) and 800-ms (dashed line) audiovisual beat stimuli for dancers (black line) and non-dancers (red line). Topographical maps showing N1 and P2 component activity under each condition. For the N1 component, the time window is set from 100–170 ms (red), and for the P2 component, it is from 170–250 ms (green). Bar colours represent amplitude intensity.

The ERP analysis enabled us to investigate the early processing of neural activity evoked by each beat (Fig 3). The ANOVA revealed a significant main effect of the beat interval on the N1 amplitude [*F* (1,61) = 32.620, *p* < 0.0001, *η*_p_^2^ = 0.348], which was significantly larger with the 800-ms beat interval than with the 400-ms interval (*p* < 0.0001). No significant interaction between the group and beat interval [*F* (1,61) = 1.069, *p* = 0.305] or main effect of group [*F* (1,61) = 0.302, *p* = 0.584] was observed. The beat interval also had a significant main effect on the N1 latency [*F* (1,61) = 3.984, *p* = 0.050, *η*_p_^2^ = 0.061], which was significantly faster with the 400-ms beat interval than with the 800-ms interval. No significant interaction between the group and interval or main effect of group was observed.

#### P2 component

The ANOVA revealed a significant main effect of the beat interval on the P2 amplitude [*F* (1,61) = 48.111, *p* < 0.0001, *η*_p_^2^ = 0.441], which was significantly greater with the 800-ms interval than with the 400-ms interval (*p* < 0.0001). No significant main effect of the group or interaction between the group and beat interval was observed. The P2 latency was not affected by the beat interval [*F* (1,61) = 0.013, *p* = 0.911], group [*F* (1,61) = 0.610, *p* = 0.438] or interaction between these variables [*F* (1,61) = 0.658, *p* = 0.420].

### Correlation analysis results

The correlation analysis revealed significant relationships between behavioral performance and neurophysiological responses across separate group analysis and the combined data analysis. For dancers, there was a significant positive correlation between tapping mean asynchrony and N1 component amplitude at the 800-ms beat interval (*r* = 0.430, *p* = 0.014). This indicates that dancers with higher tapping asynchrony exhibited higher attention allocation. Whereas for control group, there was a significant positive correlation between tapping mean asynchrony and P2 component amplitude at the 800-ms beat interval (*r* = 0.456, *p* = 0.010). At the 800-ms beat interval, significant positive correlations were found between tapping mean asynchrony with N1 component (*r* = 0.274, *p* = 0.030) and P2 component (*r* = 0.372, *p* = 0.003) amplitude when combining the data from both groups. This suggests that more accurate tapping performance demonstrated less attentional resources. No significant correlations were observed for the other conditions.

## Discussion

Studies on SMS in individuals with dance experience, compared to those without music and dance experience, is relatively scarce. In this study, synchronous auditory (pure tone) and visual (blue dot) rhythmic stimuli were used to examine the behavioral and neural activity characteristics of dancers and non-dancers during a finger-tapping task. Although the dancers did not exhibit a significant SMS advantage in response to audiovisual stimuli, they demonstrated less average asynchrony and more neural oscillation during SMS. Additionally, the beat interval significantly affected participants’ synchronization performance, which was superior with a 400-ms interval than with an 800-ms interval. Notably, the 400-ms interval was associated with faster detection and lower attentional resource.

The integration of visual and auditory information enhances perceptual–motor coordination [13]. During the audiovisual SMS task applied in this study, dancers exhibited less average asynchrony than did non-dancers, approaching synchrony with the 400-ms beat interval. However, absolute asynchrony did not differ between groups, reflecting the lack of a significant behavioral advantage in movement synchronization, although the mean value was smaller for dancers than non-dancers. Several factors could contribute to this discrepancy. Firstly, the nature of the finger-tapping task itself may not fully capture the complex SMS capabilities developed through dance training. Previous studies on SMS in dancers have employed various paradigms that differ notably from the current study. For instance, Miura and colleagues [43] used similar finger-tapping task to examine the auditory-motor coordination skills among various dance skill participants, where suggest that the skills of accomplished dancers lie in their small finger movements and that the sensorimotor learning of street dance is characterized by a stabilization of the coordination patterns compared to non-dancers. Another study also recruited street dancers showed superior whole-body SMS ability than non-dancers [22]. In contrast, our study employed a multisensory (audiovisual) task with two fixed beat intervals (400ms and 800ms). The use of simpler and fixed intervals might not have been challenging enough to reveal the potential advantages of dancers’ training in SMS. While finger-tapping tasks are a common method for assessing SMS, the characteristics of SMS among dancers, especially for different type of dance experience, should be further explored. Additionally, in studies of SMS across different modalities, people generally demonstrate optimal SMS performance in response to audiovisual beat stimuli [5, 7]. This factor could explain the lack of a significant difference between groups in the main study. Future research could explore these possibilities by employing a variety of SMS tasks, including those that more closely resemble actual dance movements, and by using more diverse samples of dancers.

Rhythm, or beat, is not an inherent physical property of music, but rather a perceptual phenomenon [44]. Broadly speaking, beat perception involves the extraction of rhythmic characteristics from the temporal attributes of external stimuli. It has two cognitive components: the perception of regular groupings, such as the familiar 4/4 beats in music or pauses between sentences in language, and the perception of isochronous pulses, such as a consistent tempo in music or steady movements in dance [1, 45–47]. Beat perception is a fundamental skill that enables individuals to synchronize their movements with the external environment and perform basic activities like walking [45, 48]. In this study, participants in both groups exhibited significantly less absolute asynchrony and better perceptual synchronization performance with the 400-ms beat interval than with the 800-ms interval, suggesting that the former was more conducive to synchronization stability. This might be attributed to the innate human rhythm preference, which is approximately 400 ms or about 120 beats per minute. Such a preference for rhythm could explain the increased accuracy at this interval [49]. Such stability has been found to decrease when individuals deviate from synchrony with beat stimuli and attempt to correct the error, particularly with longer-interval stimuli [12]. Our results further support the notion that the beat interval significantly influences beat perception and synchronization. The synchronization between neural oscillations and external rhythmic stimuli is another influential factor in SMS [15]. The dynamic attention theory posits that individual attention, functioning as an internal oscillating mechanism, can be drawn to the periodicity of sensory rhythmic stimuli, enabling the anticipation of forthcoming events [50, 51]. Consequently, the processing of beats may be enhanced during synchronized and coordinated movement.

Although our behavioral results did not show significant differences between dancers and non-dancers in terms of tapping asynchrony and stability, it is important to consider the neural data. Compared with the 400-ms beat interval, the 800-ms interval induced significantly more brain neural activity, eliciting more synchronized neural oscillation responses to the external beat, in this study. Thus, the external beat frequency influences oscillatory activity during SMS. Compared with the 400-ms beat interval, the 800-ms interval is relatively uncommon in daily life. In this study, participants performed synchronous responses while perceiving rhythm frequency synchronization. Longer beat intervals may require a greater allocation of attentional resources and result in higher-power allocation during synchronization, explaining the greater neural activity associated with the 800-ms interval. This finding may also be attributed to the inherent characteristics of low-frequency oscillations, characterized by longer periods and greater amplitude. This resonant behavior at longer intervals aligns with the notion that low-frequency neural oscillations exhibit greater energy at slower temporal rates. Moreover, significant differences in power at the frequency of 1.25 Hz were observed between dancers and non-dancers, with the former exhibiting more pronounced and synchronous neural oscillatory activity. The delta band results also confirmed the significant effects of beat frequency and dance experience, with more power observed with the 800-ms interval than with the 400-ms interval and among dancers relative to non-dancers. These findings demonstrate that the beat frequency is a primary determinant affecting neural activity, and that the heightened neural oscillation observed in dancers is an internal factor contributing to their superior beat perception relative to non-dancers.

Audiovisual beats provide subjects with auditory and visual attentional information, and the engagement and allocation of attentional resources may impact SMS [52]. In this study, the N1 amplitude and latency induced by the 800-ms interval were significantly greater than those induced by the 400-ms interval, suggesting that the longer interval generated a greater demand for attentional resources and allocation during the early processing stages, likely due to the increased difficulty of action synchronization. The P2 component induced by the audiovisual beat, reflecting a later stage of attentional processing, also demonstrated the influence of the beat interval on action synchronization; the P2 amplitude was greater with the 800-ms interval than with the 400-ms interval, reflecting greater allocation of attentional resources. Interestingly, our findings revealed 400 ms condition despite showing reduced behavioral variability, was associated with smaller ERP amplitudes. The reduced standard deviation and smaller ERP components observed under the 400 ms condition can both be related to the human preference for certain rhythms [49]. In conditions with familiar and simpler rhythms, synchronization performance tends to be better and more stable, and the cognitive resources required are lesser. This could be a plausible explanation for these results. In familiar rhythm conditions, participants might require less cognitive effort, leading to both reduced variability in performance and smaller ERP amplitudes, reflecting a more efficient neural processing. These results revealed greater resonance power in delta band frequencies in dancers and significant differences in ERP components (N1 and P2) between the 800-ms and 400-ms beat intervals, suggesting that while overt performance may be similar, the neural mechanisms underlying this performance differ between groups. This supports the hypothesis that dancers may engage different or more efficient neural processes when synchronizing to rhythmic stimuli.

Previous study quantified neural entrainment in an auditory-motor synchronization task using EEG data [53]. The results showed the stability index was significantly and negatively correlated with key behavioral measures, indicating its role as a reliable neural marker of entrainment. Whereas, in the present study, the correlation analysis reveals distinct group-specific relationships between behavioral performance and neurophysiological responses during the 800-ms beat interval. For dancers, a positive correlation between tapping asynchrony and N1 amplitude suggests that greater attentional resources are linked to higher asynchrony. In contrast, non-dancers showed a positive correlation between asynchrony and P2 amplitude, indicating a different neural response mechanism. The overall significant correlations between asynchrony and both N1 and P2 components underscore the role of attentional modulation in SMS, particularly under challenging rhythmic conditions. These findings suggest that dance experience influences the neural strategies employed during SMS and underscore the need for further research to fully understand these dynamics.

However, there are some limitations in the present study. While our study provides valuable insights into SMS using the finger-tapping task, it’s important to acknowledge the limitation that this method primarily captures synchronization at a fine motor level. The absence of significant behavioral differences might be attributed to various factors such as sample size, task difficulty, or individual differences within groups. The discrepancies between our findings and previous studies highlight the importance of considering the specificities of the experimental paradigms and the characteristics of the dance training when investigating SMS in dancers. Future studies should aim to systematically vary task complexity, sensory modalities, and movement types to better understand the conditions under which dance training enhances SMS performance.

## Conclusion

In conclusion, our study using an audiovisual SMS task highlights the complex interplay of beat interval, synchronization performance, and neural oscillatory activity. Both dancers and non-dancers exhibited better synchronization performance and stability at the 400-ms interval compared to the 800-ms interval. No significant main effect of the group was identified, indicating that the observed performance pattern was consistent across both dancers and non-dancers in audiovisual beat condition. These results suggest that synchronization performance is influenced by the rhythm frequency, with shorter intervals (400 ms) facilitating more accurate and stable synchronization in both groups. Our findings indicate that longer beat intervals pose increased task difficulty and demand more attentional resources, which may affect action synchronization and detection speed in SMS. This study contributes to the understanding of rhythmic processing and its neural correlates, and underscores the need for further research to explore the influence of training and experience on rhythmic perception and synchronization. Future studies could extend this work by examining the effects of varying levels of expertise and training on rhythmic processing and neural synchronization, especially bridge the gap between simplistic laboratory tasks and the complex, multi-dimensional nature of real-world dance movements, providing deeper insights into the neural mechanisms underlying beat perception and coordination.

## Supporting information

S1 Dataset The raw data supporting the conclusions of this article are available in the supplementary materials. (XLSX)
